# Dental Hints Department

**Published:** 1907-09

**Authors:** B. H. Teague

**Affiliations:** Aiken, S. C.


### ...DENTAL HINTS DEPARTMENT...

CONDUCTED BY B. H. TEAGUE, D. D. S., AIKEN, S. C., TO WHOM ALL CONTRIBUTIONS TO THIS DEPARTMENT SHOULD BE SENT

The following is a formula for sticky wax: Pure white beeswax 4 oz., pure light yellow resin 7 oz., pure gum dammar 1 oz., dye q. s. to color. Powder the resin and gum dammar, and add little by little the melted beeswax.—Dental Record.

For neuralgia: Tanic acid 2 parts, rectified spirits 10 parts. Rub on part affected.—Exchange.

Sterilizing, Polishing and Grinding Stones.—Brush clean with soap and water with a plate brush, and then dip in a 5 per cent. solution of formalin in alcohol.—Tri-State Dental Record.

An anodyne to prevent soreness after removing nerve pulp: ℞. Menthol, ʒjss; thymol, ʒijss; phenolis, ℥j. Mix and use as indicated.—Dr. J. P. Buckley, Review.

Arrested.—Three dentists in Pittsburg who had been working in a dental parlor were arrested for practicing without a state license. They were graduates of dental colleges, but had not passed the state board.—Exchange.

To Allay Pain After Extracting:—℞. Orthoform Europhen, aaʒj; liquidi petrolati q. s. to make a paste. Mix. Apply to socket and abraded surface.—Dr. J. P. Buckley, Review.

To Prevent Nausea:—When administering morphine internally to one-fourth grain of morphine add in water 20 grains of Bromide of Potash.—Dr. G. W. Rembert, New Orleans, La.

RUBBER VARNISH:—A small per cent of Spts. Turpentine added to base plate rubber dissolved in chloroform, will cause it to stick better and not peel from plaster cast.—DR. J. B. BIGHAM, Chester, S. C.

Application for nerve canals when fitting with gutta percha. ℞. Menthol, gr. iij; thymol, gij; encalyptol, f℥j. Mix. Dress nerve canal before inserting point.—Dr. J. P. BUCKLEY, Review.

ARSENICAL PASTE.—℞. Arseni trioxidi, ℥j; cocaine hydrochloratis, grxx; menthol, grv; lanolini, q. s. to make a stiff paste. Mix. Use desired quantity. Color with lamp black.—DR. J. P. BUCKLEY, Review.

A piece of a common brick is a good thing to rub an instrument on to remove rust. Oil it and rub it in the same spot. The rough surface will first remove the rust and as the spot becomes smooth it will polish the instrument.

CHLORAL HYDRATE.—Apply a crystal of chloral hydrate to an aching pulp and cover with soft gutta-percha to stop the pain. For pericementitis, make a solution of one part chloral hydrate in five parts of glycerine and paint over the parts involved.—DR. C. L. CUINCEROT, Cosmos.

REMEDY FOR A WARPED PLATE.—A rubber plate which has been bent out of shape will return to its original shape if dropped in hot water and kept there a few moments.—Pacific Dental Gazette.

TO PREVENT ETCHING.—To prevent "etching" of facings when using twenty per cent platinum solder, paint the surface with a creamy solution of magnesium carbonate before investing.—DR. A. E. MANESON, Review.

DAM A TOOTH AND INLAY IT.—Dr. Kirk is quoted as saying, "If a tooth is worth a dam, put it on." Very good advice at the time it was given, but when my dentist tells me one of my teeth is worth a dam, I say, put in an inlay!—DR. C. S. VAN HORN, Cosmos.

Blue Rays.—In view of the several uses to which the blue ray is already being put, is it too much to prophesy that the day is not very distant when this modest agent will all but revolutionize dental therapeutics?—when, indeed, it will have done even more for our profession than the Roentgen ray has for general therapeutics?—Dr. W. J. Hodson, Cosmos.

Prophylaxis.—The D. D. Smith idea of prophylaxis I have followed for a period of nine years and it is the greatest means of preventing tender gums and decay of teeth that has ever been suggested. A mouth can be made practically immune from decay.—Austin F. James, Western Dental Journal.

To Prevent Rubber Warping on Aluminum Plates.—When the base-plate is made and spurred, if instead of using the ordinary vulcanite and placing it in contact with the metal, you will put on a thickness of what we call weighted rubber, you will find it will persist in hugging and will not warp away, as the pure or straight vulcanite does.—R. C. Brophy, Dental Era.

A Big Question Mark.—I put a big question mark after the statement that the intermaxillary rubber ligatures are effective for retention. What are you doing while holding those rubbers on the teeth? You certainly will not expect a stationary retention or even rest. It is a serious question, and that kind of anchorage is a confession of a failure in retention and regulation.—Dr. V. E. Barnes, Items.

Painless Tumors Dangerous.—A tumor situated in the mouth, or more particularly in the gum, alveolar process, or jaw bones, which is not painful, is not accompanied by inflamation, and which grows rapidly, should be observed with the utmost suspicion because such tumors are, as a rule, malignant.—Dr. W. T. S. Dodds, Items.

Cause of Constipation.—One great source of constipation is lack of water in the system. The feces become dry because the water in the intestinal canal is absorbed. This may be demonstrated by measuring the quantity of urine passed every twenty-four hours. The normal quantity should be three pints or forty ounces. That much water should be drunk each day including tea, coffee and milk.— E. S. TALBOT, American Dental Journal.

FIVE HOURS PRACTICE ENOUGH.—I still believe that the average dentist will make more money, live a longer and more useful life by practicing from ten to three continuously, and devoting the remaining part of the day to healthful, recreative pursuits, baths and massage, golf, tennis, driving, etc., than to put in the laborious hours which many do, and which seem momentarily profitable, but which with the great majority lead to mental depression, general ill-health, and early decay.—WM. LEON L. ELLERBECK, Cosmos.

TRY YOUR FURNACE.—In using any open-mouth furnace, to determine the exact state of fusion of porcelain, while baking, take a burned match, hold in furnace tongs (a pair of scissors handle artery forceps make good tongs), open furnace to light match, the contrast flame from the pine will enable you to see the exact state of fusion. Try it. Do not use a match that has not been struck, the sulphur is objectional. Also remove the carbon from match. Lead before inserting in furnace.—H. H. SULLIVAN, Western Dental Journal,

SENSITIVE DENTIN.—Some time ago Dr. Fossume spoke of the use of "Formalin" for controlling the sensitive spots at the necks of the teeth that are sometimes so troublesome. At that time I questioned its safety, but since trying the method I have found it so satisfactory that I wish to speak of it at this time. The "Formalin" should be used full strength and it must be kept away from the gum. A very short application is sufficient to control entirely a very large proportion of these cases.—H. W. GILLETT, Journal.

ROOT CANAL FILLING.—After pulp removal under pressure anaesthesia I always leave a small quantity of mummifying paste (zinc oxid, alum and thymol) at the extreme end of the canal and then fill with gutta-percha points dipped in a saturated solution of thymol in oil of cinamon. The cinamon gradually evaporates, leaving a layer of thymol crystals, lining the root canal.—WILFRED E. GRIFFIN, British Dental Journal.

ABNORMAL OCCLUSION IN TEMPORARY TEETH.—I think there can be no question but that there are large numbers of cases where the temporary teeth are not in normal occlusion. I have observed many myself, and Dr. Bogue has shown some on the screen. I have had two cases called to my attention since I have been in this city of complete distal occlusion in the deciduous teeth. In those cases the first molar will hardly be guided into its normal position.—DR. J. B. LITTIG, Items.

ANTRAL EMPYEMA.—In all cases of acute or chronic antral empyema, also with cases of clear dental etiology, it is the duty of the dentist to require the diagnostic help of the rhinologist and to secure the investigation of possible nasal etiological factors or nasal complications. Without determining this, the dentist runs the risk of never curing uncombined antral empyema.—HERMAN STOLTE, M. D., Review.

LICENSE EXCHANGE.—The importance of the subject of license exchange between states is such that any man, whatever his standing, should deem it an honor to do something toward equalizing the standing of dentists in the various states. Please don't feel satisfied that you will never need a change of location. It may pinch you as it has others.—F. R. HOUSTON, Review.

HYDRONAPHTHOL AS A PULP-CAPPING.—To avoid the removal of the layer of softened detine, which if removed, would probably necessitate the removal of the pulp, mix equal quantities of hydronaphthol and cement, and place as a capping on the layer of decalcified dentine, allowing it to set. Then proceed with the filling. The hydronaphthol arrests bacterial action.—A. W. MCCALL, Federal Dental Journal.

Early Symptoms of Autointoxication.—The first symptoms of pathologic effects due to autointoxication are observable in the alveolar process. In the treatment of interstitial gingivitis, the stomatologist should be mindful of the fact that the patient's system is tending toward disease—E. S. Talbot, Dentists Magazine.

Cement Fillings.—When conditions indicate the advisability of having a small portion of infected (though not softened dentin) the cavity must be sterilized with a cauterizing, non-staining disinfectant, such as colorless tricresol and formalin, equal parts; all traces of this solution to be removed from the cavity by applications of hot air, or repeated washings with distilled water, before the cement is inserted.—R. H. Welsh, Items of Interest.

Independent Dental Mariners.—Many men there are in the dental profession today who have been given a license to practice who have cut the hawser that bound them to the school of progress. They have, it is true, chart, compass and chronometer on board, but they have said, "I will sail my own bark. I recognize no authority above my own, no responsibility to those in my care. The goal of my ambition is a place over there some twenty, thirty or forty odd years away where there are sufficient dollars to keep me in ease and comfort until this life is ended," and they act as if this life ended all.—Dr. T. F. Cooke, Dentists Magazine.

Amalgam Fillings.—For amalgam fillings in first and second molars, gold is indicated to bring the alloy to the required hardness and edge strength. If copper is substituted instead of gold the result is rapid discoloration, whereas gold whitens the alloy and hardens it to any desired extent, and most important of all, gives such a knife-edge water-tightness as seems to meet with no equal.—W. Charles Davis, The Dental Record.

Extreme Commercialism not Professional.—No man has a right to remain in any profession or any calling who has just one object in view, and that object—obtaining what money he can out of others. The best there is in a person is not brought out if money is the goal. If there can be no display of heart in what is being done, I fear that there is not very much pleasure for the man who operates.—Dr. E. K. Wedelsteadt, American Dental Journal.

A Way to Prevent Pain in Filling Children's Teeth.—Where children come with large cavities in molars, but with no pulp exposure as yet, the thorough and often painful (painful if thorough) removal of decay may be avoided if the nitrate of silver treatment is used prior to inserting a filling; and thus our work, is durable, while tediousness and pain are largely eliminated, and tho child is our friend.—Dr. R. B. Tuller, American Dental Journal.

Do You Advise Weighing the Mercury and Alloy for Each Mix?—I do not. The secret of success lies strictly in the amount of mercury that you are capable of removing from the mass rather than the amount that you use when first forming the mix. It is vitally important to use a surplus of mercury when first forming the mix in order that you may dissolve every particle of alloy. The integrity of the mix may be preserved by squeezing the surplus of mercury through drilling or chamois skin. The most important metals belong to the "crystalline group," and are not removed from the mass during the wafering process.—N. K. Garhart, Western Dental Journal.

Unfilled Canals.—I have filled upward of four thousand, I will not say pulpless teeth (I have tried to have them pulpless), but devitalized teeth, and in doing so have used almost every known method, and I am frank to confess that the percentage of failures with the so-called permanent canal filling and those so-called temporary dressings containing a medicament so applied as to make removal and retreatment easy, are about equal; but because of the difficulty of removal of the one and the easy removal of the other, the difficult ones seem to outnumber the others as ten to one.—Dr. W. E. Tuer, Items.

Soft Amalgam.—Never leave a soft mass of amalgam on the surface of the filling; scrape it out and pack in harder amalgam. Leave the fillings over full and when the amalgam has set dress it to the proper contour. Shrinkage, spheroiding and change of form are the three greatest amalgam faults; all of these are due to faulty manipulation of the amalgam in mixing. The high grade alloys do not amalgamate easily, hence care should be used to produce perfect mixtures. I get the best results by using enough mercury and expressing all excess carefully and thoroughly.—Dr. N. K. Garhart, Register.

Method of Taking Plaster Impressions in Partial Cases.—I take an impression without an impression cup. In doing this the first thing is to have the patient rinse the mouth with milk of magnesia. This lubricates the teeth and makes it possible for the plaster to get a close adaptation to the teeth, without sticking to them. If the plaster is mixed so that it will not drop off in chunks, but run off, you can keep on piling up until you have got sufficient surface, so that you will have as much bulk of plaster as you would in an impression cup. After the plaster has become hard it will not readily move out of place, but may easily be cut with a spatula. I slit it half way through in three or four places, and with a broad spatula placed in the full length of the slit, twist it out and spring it apart. The pieces may easily be placed together again after removal.—William H. Taggart, Review.

"Tc Maim His Patient For Life."—This is a fundamental truth that should be indelibly impressed upon the mind and conscience of every dentist whether he be an operator, prosthetist, orthodontist or surgeon. He is maimed for life, both from the viewpoint of usefulness and cosmetics. The patient can use only about one-fourth the comminuting force on a denture resting upon the mucous tissues that he can upon the natural teeth. Because of the decreased curve of the summit of the alveolar process in an upper edentulous jaw, and, for the concentration of energy in mastication, the molars and bicuspids of the artificial denture are made much smaller upon the occlusal surface than the natural teeth; thus, much time is required to properly insalivate the food; hence the patient is robbed of many hours each year that he might apply to work or recreation, else indigestion and a shorter life are invited by imperfect mastication.—Dr. F. K. Ledyard, Dentist's Magazine.

Exchange of License.—A peck is a peck in all States; a pint is a pint, and a pound is a pound. All miles are of the same length, and all acres have the same dimensions. But the measure of a dentist varies in all parts of this country; hence the fallacy of declaring that a dentist good enough for one state is good enough for all. Undoubtedly there are dentists in all states capable of practicing in any state. But passing to the opposite extreme, there are dentists practicing in all states that are not fit to practice in any state, which shows the defects in the methods of measurement, and the error of demanding interchange of all licenses, without restrictions.—Items.

Ring a Root for a Logan Crown.—It is good practice when using the Logan Crown regular to insert a ring or band with quick-setting cement, and grind even with the face of root then set your crown as in other cases. This will prevent the root from splitting on account of undue lingual pressure.

The advantages of the method are no irritation to soft tissues due to an ill-fitting band, no metal visible and universal application to all porcelain or porcelain combined with metal.—Dr. S. D. Ruggles, Register.

To Be Worn How Long?—There seems to be no well defined idea as to how long a so-called permanent denture should be worn. It is the opinion of the writer that the best interests of the patient would be subserved if they did not wear any denture more than five or six years, unless upon examination it should be found there was perfect adaptation, and the base plate was of a non-absorptive material. * * *

As there are only a few dentists limiting their practice to prosthesis, it follows that most of the prosthetic restorations must be made by the general practitioner. The general practioner is the operator, prosthetist and extractor combined in one, and his duty to his patients is always in the order named. His first and greatest duty is to save all teeth possible; his second duty is to restore lost teeth in as useful and esthetic a manner as possible; and his third duty is never to extract a tooth if he can avoid it.—Dr. G. H. Wilson, Dominion.

Advice to Patients.—I care not how skillful a practitioner is, he cannot build or retain a practice if he does not handle his patients right. I have known convincing talkers lacking in dental skill to be more successful than some of the very best men in the profession. One of the first things I do when patients come to my office for a set of teeth is to explain to them that there are always two sides to a contract, and that they are under just as many obligations to me as I to them. They are expected to give their very best efforts as well as me. That it is unnatural to wear artificial teeth, and that it must be learned as well as any other accomplishment, such as playing on the piano, skating or swimming, and if they will agree to give the teeth a fair trial, allowing me to be the whole judge, that I will take the case.—Dr. O. H. Simpson, Western.

Where Gold Inlays are Indicated.—Gold inlays are indicated in the following places:

In large cavities in teeth of children.

In cavities in teeth that are attacked by chronic interstitial gingivitis or pyorrhea.

In cavities in the teeth of very nervous people or those the muscles of whose jaws are affected by cramps.

In cavities that are inaccessible either on account of a small opening to the mouth or unusual position of the teeth. Accessibility should be the guide in many cavities whether to make a gold foil filling or an inlay.

In cavities the walls of which are too weak to stand the malleting necessary for gold foil and which are not so far gone as to necessitate crowns. This will include many teeth which in former years it would have been necessary to crown, but which in the hands of the experienced inlay worker can be made to do years of service and with a much more healthy environment than would have been the case had they been fitted with crowns.—Dr. Chas. E. Woodbury, Register.

Alphazone.—For some time past I have been using Alphazone with success, as a disinfectant in place of hydrogen peroxide. Alphazone is an organic peroxide, but, unlike other peroxides, it does not effervesce and cause pain when sealed in a cavity. Although it has been proven to be many times stronger in its germicidal action than phenol, formalin or cresol, it does not irritate the tissues of the mouth. It is particularly useful in cases where it is impossible to place a dam. In these cases, the canals and the whole mouth as well, can be washed thoroughly with the solution. It is slightly acid, and leaves a faintly metallic taste. Most patients do not even speak of the taste. It is not stable after two or three days, but a one one-thousandth solution is available almost as soon as a pellet is dropped in two oz. of water, so it is easily made fresh each day.—Dr. L. P. Hall, Register.

To Replace Tooth on Bridge.—The first thing in repairing a bridge, I take a facing suitable to the case in hand. I drill my holes through the backing, and adjust the teeth in the proper position. Then I have a thin sheet of platinum—there is less danger of burning by solder than gold. Take a pair of pliers. Rub that around the tooth-pin; it will form a tube. Then clip off the pin from the tooth; close up the tooth; put the smallest particle of solder on that with a little borax; bed the tooth in the compound composed of asbestos and Portland cement—I buy it from hardware stores; they use it for covering plumbing. I do not wet the investment, but simply heat it up. Then you have a tooth with two hollow pins in it. Enlarge the hole slightly in your backing. Adjust your tooth. Use your cement if necessary. I use a ball-headed burnisher to spread the tube on the lingual end, and I have an attachment somewhat similar to the attachment on the eyelet in a shoe. It makes a small hole there, which if you are using platinum you can fill with amalgam or with gold. If it is inaccessible you should use amalgam; if it is accessible it is preferable, of course, to use gold. You can hardly tell that the tooth has been replaced. I have been doing that for some years. So far I have found it entirely satisfactory.—Dr. W. A. Burns, Dominion.

Root Amputation.—It has been my practice in treating chronic abscesses, when they do not yield to treatment readily, to use a local anesthetic and amputate the roots of the teeth. I do not mean all three roots of an upper molar if it is not necessary, but where you can amputate one or two roots of these, or one root of an inferior tooth, this treatment will result most satisfactorily in many cases. How often have you filled teeth where you have had chronic abscesses treated for several weeks, and in six weeks the patient returned with a recurrence of the condition worse than before. If you had gone through the process with a sharp burr and satisfied yourself that you have a carious or necroses condition of the bone, where medicine cannot effectually be employed, and surgically treated it you would have a permanent result.—Dr. Oakman, Register.

Amputating Roots.—I think it is not as we do it so formidable as many of us would think. It is not necessary that we actually cut in there and remove part of the root; often it is only a certain root that is necrosed, and if there is any portion of the root diseased, it can be removed very easily with a burr. So far as amputation of the roots is concerned, this is very seldom necessary in my experience, but I do not hesitate if after a few treatments by the latest and most improved methods, and the tooth does not get better, and if I have found that the tooth canal is aseptic enough, I usually fill that root canal, then by means of phenol and cocaine I open up and remove all the necrotic tissue that I find surrounding the apex of that root and the necrotic tissue, being very much softened, will be removed, and the healthy, hard tissue will not yield to the burr. It is often only necessary to remove the necrotic tissue and not any great amount of the roots. However, if you have a larger necrosis followed by disintegration of the tissue you have to remove part of the root, and its removal, of course, is followed very often by very good results.—Dr. Graham, Register.

Artificial Dentures Should Duplicate Nature.—A full upper and lower denture should, if correctly constructed, duplicate the conditions found in Nature. That is, there should be numerous and widely distributed points of contact between the two sets of teeth, so that when the movements incident to mastication are performed, the two sets will be held in place, and there will be no tipping of the plates upon the alveolar ridges. In setting up the teeth, this condition is more easily arrived at, if the process of eruption of the natural teeth is kept in mind and imitated. The appearance of the plate in the mouth and its harmony with the features, depends almost entirely upon the arrangement of the upper incisors, cuspids and bicuspids, and it is always advisable to try the upper plate in the mouth of the patient after these teeth have been arranged and before proceeding further. When assurance is had that these teeth are correctly set, the lower second bicuspids are next mounted so that their cusps come into the interspace between the upper bicuspids. The lower first molars follow, their height being gauged by the overhanging distal surface of the upper second bicuspids; and their positions are tested by lateral movements of the articulator, and they are set so that they will maintain contact, but not interfere with that of the bicuspids. Then the upper first molars are added, and their positions tested in the same manner. After these come the lower second molars and the corresponding upper teeth. When they are all mounted, they should all remain in contact during lateral movements of the articulator.—Dr. G. B. Snow, Dentists' Magazine.

